# Tracing the Origin and Evolutionary Fate of Recent Gene Retrocopies in Natural Populations of the House Mouse

**DOI:** 10.1093/molbev/msab360

**Published:** 2021-12-23

**Authors:** Wenyu Zhang, Diethard Tautz

**Affiliations:** Department of Evolutionary Genetics, Max Planck Institute for Evolutionary Biology, Plön, Germany

**Keywords:** gene retroposition, house mouse, natural populations, adaptation, population divergence

## Abstract

Although the contribution of retrogenes to the evolution of genes and genomes has long been recognized, the evolutionary patterns of very recently derived retrocopies that are still polymorphic within natural populations have not been much studied so far. We use here a set of 2,025 such retrocopies in nine house mouse populations from three subspecies (*Mus musculus domesticus*, *M. m. musculus*, and *M. m. castaneus*) to trace their origin and evolutionary fate. We find that ancient house-keeping genes are significantly more likely to generate retrocopies than younger genes and that the propensity to generate a retrocopy depends on its level of expression in the germline. Although most retrocopies are detrimental and quickly purged, we focus here on the subset that appears to be neutral or even adaptive. We show that retrocopies from X-chromosomal parental genes have a higher likelihood to reach elevated frequencies in the populations, confirming the notion of adaptive effects for “out-of-X” retrogenes. Also, retrocopies in intergenic regions are more likely to reach higher population frequencies than those in introns of genes, implying a more detrimental effect when they land within transcribed regions. For a small subset of retrocopies, we find signatures of positive selection, indicating they were involved in a recent adaptation process. We show that the population-specific distribution pattern of retrocopies is phylogenetically informative and can be used to infer population history with a better resolution than with SNP markers.

## Introduction

Gene duplication is a major mechanism to generate adaptive evolutionary novelties in organisms (Ohno 1970; Long et al. 2003). Gene retroposition (or RNA-based gene duplication) is a particular type of gene duplication in which a gene’s transcript is used as a template to generate new gene retrocopies, and this has a variety of evolutionary implications (Kaessmann et al. 2009; Casola and Betran 2017). Despite of inherent lack of the ancestral regulatory sequences, abundant previous between-species comparison studies have found a substantial number of new gene retrocopies acting as functional retrogenes that encode full-length proteins across organisms (Bai et al. 2007; Baertsch et al. 2008; Kaessmann et al. 2009; Casola and Betran 2017), via recruiting regulatory elements from the vicinity or evolving them de novo. Also, given that the majority of genomic regions are accessible to transcription (Neme and Tautz 2016), many of the inactivated gene retrocopies with truncation (i.e., processed pseudogenes) could also be transcribed, and thus their transcripts could influence the expression and function of the parental genes (Tam et al. 2008; Marques et al. 2011). In addition, the distinct patterns on the extensive gene traffic on the X chromosome indicate that the fixation of most of the functional retrogenes is driven by positive selection (Betran et al. 2002; Emerson et al. 2004). Although these between-species comparison studies have provided important insights into the functional evolution of gene retrocopies at a long-term evolutionary time scale, the evolutionary dynamic and trajectory of newly arrived gene retrocopies, especially at the very early stage when they are still polymorphic within natural populations, remain largely unexplored.

The accumulating population genomic sequencing data allowed to study polymorphic gene retrocopies (retroCNV alleles or retroCNVs) at individual genome level within natural populations. Through analyzing the genomic sequencing data set of natural samples from the 1000 Genomes Project Consortium (1000 Genomes Project Consortium et al. 2012), several studies have expanded the gene retrocopy repository in human populations, and revealed substantial polymorphisms of retroCNVs among human individuals (Abyzov et al. 2013; Ewing et al. 2013; Schrider et al. 2013; Kabza et al. 2015; Zhang et al. 2017). However, studies that explore the retrocopy dynamics in the context of natural populations from different subspecies are still lacking. We analyze here the evolutionary patterns of gene retrocopies at individual genome level in house mouse populations, based on a set of well-defined natural populations from different lineages and subspecies where evolutionary processes and retroposition rates can be analyzed at a much broader comparative level than it is possible for humans.

Owing to its well-defined evolutionary history, as well as a set of largely homogeneous genomic resources from wild mice collected from nine world-wide natural populations and two out-group species (Harr et al. 2016), the house mouse (*Mus musculus*) represents a unique model system for the study of evolutionary dynamics of recently originated gene retrocopies. The inclusion of the genomic sequencing data from out-group species allows the detection of newly arrived species-specific gene retrocopies, whereas comparable population data of out-groups (e.g., Neandertals or Denisovans) is not available in human studies. Using this unique genomic data set, we identified previously more than 2,000 house mouse lineage-specific gene retrocopies, and showed that the primary rate of gene retroposition in house mouse natural populations is orders of magnitude higher than the previously estimated long-term rate (Zhang et al. 2021). Our analysis showed that the majority of them have deleterious effects, not only through disrupting functional genomic regions, but also by virtue of their expression that can interfere with their parental genes, especially when they are transcribed in antisense direction. Given the advantageous nature of the sequencing read features (e.g., sequencing coverage, read length, and insert size), our previous study revealed a much higher detection rate of insertion sites of gene retrocopies than was reported in human studies (Zhang et al. 2021). Therefore, this made it possible to use this data set to directly trace the origin and evolutionary dynamics of very recently derived gene retrocopies and their potential contribution to the adaptation process in natural populations.

We mainly focus on the possible adaptive effects of new gene retrocopies in the present report. We show that evolutionarily old house-keeping genes are the major source of gene retroposition to generate new gene retrocopies in house mouse wild individuals. Chromosomal movement analysis reveals a stepwise emergence of the “out-of-X” pattern of gene retroposition, suggesting that the action of positive selection on a subset of new gene retrocopies can result in an increase of their frequencies in populations. Positive selection signal detection analysis further shows the potentially direct action of positive selection on a subset of gene retrocopies that are private in single subspecies, hinting these gene retrocopies might play a role in the adaptation process of the respective populations. Given that the results show that retrocopies are strongly influenced by positive and negative selection in natural populations, we ask whether they are actually better suited for analyzing population differentiation processes than mostly neutral SNPs. We show that this is indeed the case, thus confirming the largely non-neutral evolutionary dynamics of newly derived gene retrocopies.

## Results

We made use of 2,025 house mouse lineage-specific retroCNVs with mapped insertion site that were identified in our previous study (Zhang et al. 2021). All of them have not reached fixation among the 96 wild caught mice individuals derived from nine natural populations, covering all the three major subspecies of the house mouse (*M. m. domesticus*, *M. m. musculus*, and *M. m. castaneus*). These retroCNVs are derived from 1,372 unique parental genes (supplementary table S1, Supplementary Material online). Only 81 (4%) of these retroCNVs are annotated as recently originated retrocopies in the mm10 reference genome based on RetrogeneDB v2 (Rosikiewicz et al. 2017) (≥95% alignment identity to their parental gene, see Zhang et al. [2021]) (supplementary fig. S1*A*, Supplementary Material online). Most of the retroCNV parental genes (71%) have only one retroCNV allele, whereas the rest are recurrently retroposed to generate multiple gene retrocopies with up to eight distinct insertion sites across individual genomes (supplementary fig. S1*B*, Supplementary Material online).

### House-Keeping Genes as the Major Source of Gene Retroposition

**Fig. 1.**
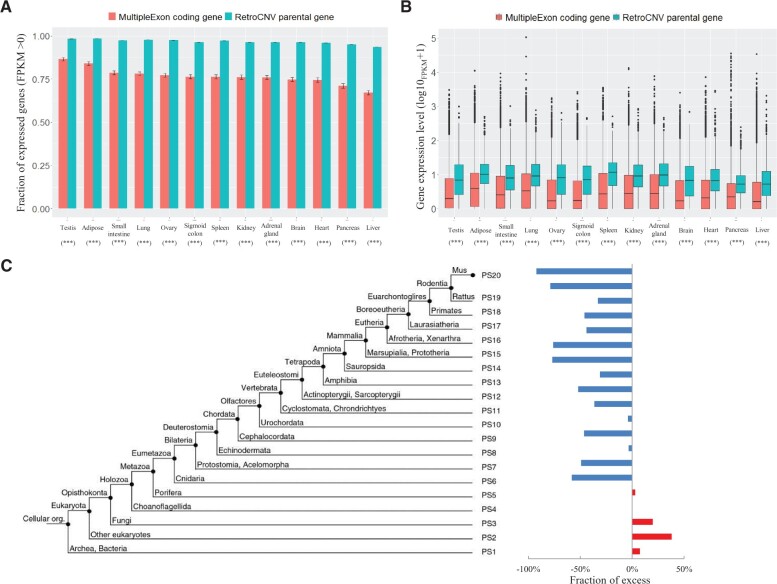
Characteristics of retroCNV parental genes. Comparison of fraction of genes with detectable expression (*A*) and expression level (*B*) between retroCNV parental genes and annotated protein-coding genes with at least two exons. The detectable expression is defined as FPKM > 0. (*C*) shows the fraction of excess of observed retroCNV parental genes in comparison with null expectation, on the basis of the distribution of annotated protein-coding genes of at least two exons within each evolutionary PS. The PS assignment data for mouse protein-coding genes were retrieved from Neme and Tautz (2013). The greater values succeeding the prefix “PS” indicate the younger gene groups. Error bars in panel (*A*) show the standard errors of average fractions of expressed genes for annotated protein-coding genes with at least two exons (*N* = 20,500), from which 100 times random sampling processes of the same gene number as that of retroCNV parental genes (*N* = 1,372) were performed. All the statistical analyses were performed by using Wilcoxon rank-sum tests. ***P value ≤ 0.001.

Through analyzing the transcriptomic sequencing data from 13 tissues of the *M.musculus* C57BL/6 inbred line (Lin et al. 2014), we found that almost all the retroCNV parental genes (98.9%) showed detectable expression in germline tissues of testis and ovary (fig. 1*A*), consistent with the prediction of a ubiquitous germline expression of parental genes (Kaessmann et al. 2009). The remainder may be expressed during germline development to enable gene retroposition events to become inherited. We found both a significantly higher fraction of genes with expression (FPKM > 0, fig. 1*A*) and a significantly higher expression level for retroCNV parental genes in germline tissues (fig. 1*B*), compared with all the annotated protein-coding genes with at least two exons as a control (only protein-coding genes with at least two exons were used for the detection of gene retrocopies, see Zhang et al. [2021]). Additionally, in-depth gene expression pattern analysis revealed that retroCNV parental genes being recurrently retroposed to generate multiple retrocopies had a significantly higher expression level in germline tissues, in comparison with the ones that were retroposed once only (supplementary fig. S2, Supplementary Material online).

Interestingly, a similar pattern on a higher expression level of retroCNV parental genes was also observed for all the tested somatic tissues (fig. 1*A* and *B* and supplementary fig. S2, Supplementary Material online). Furthermore, gene ontology (GO) enrichment analysis on these retroCNV parental genes revealed their involvement in a variety of biological processes for the maintenance of cellular function (supplementary table S2, Supplementary Material online), a typical characteristic of house-keeping genes (Eisenberg and Levanon 2003), suggesting these retroCNV parental genes tend to be house-keeping genes.

Given that the evolutionarily old genes usually harbor broader expression and higher expression levels in multiple tissues (Zhang et al. 2015), one can speculate that retroCNV parental genes should be enriched in evolutionarily older gene groups. To test this hypothesis, we calculated the fraction of excess of observed retroCNV parental genes with null expectation, on the basis of the distribution of annotated protein-coding genes with at least two exons (same as above), for which the gene age assignment data of mouse protein-coding genes was retrieved from Neme and Tautz (2013). Our results indeed showed a positive excess of retroCNV parental genes that originated before Metazoan, whereas a consistent negative excess can be observed for all the evolutionarily younger gene groups after that split (fig. 1*C*). The analysis based on another independent gene age assignment data from Shao et al. (2019) revealed the same pattern on the enrichment of retroCNV parental genes on evolutionarily older gene groups (supplementary fig. S3, Supplementary Material online). These findings further support the conclusion of house-keeping genes as the major source of gene retroposition.

### Indicators for Positively Selected Gene Retrocopies

Most newly originated gene retrocopies are deleterious and are quickly selectively purged (Zhang et al. 2021), but a fraction of them show higher frequencies in natural populations, either because they are neutral and have accumulated through drift effects, or they have a beneficial role and rise in frequency due to positive selection.

**Fig. 2.**
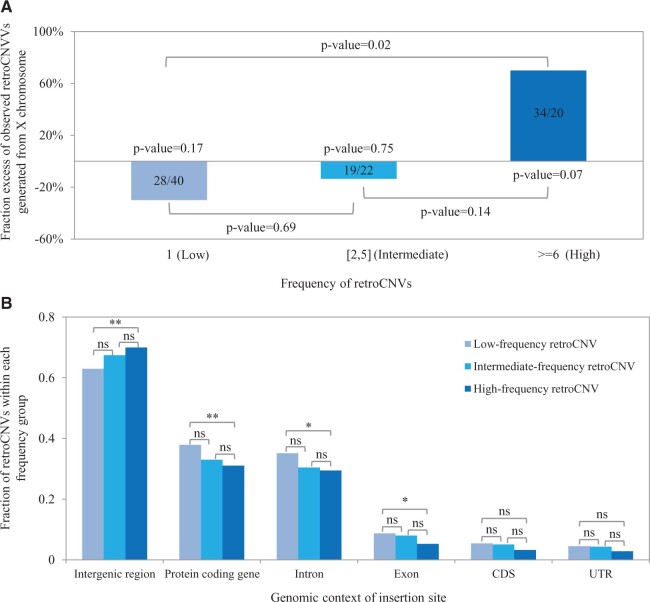
Features of retroCNVs from different frequency groups. (*A*) Fraction excess of observed retroCNVs with each frequency group generated from the X chromosome. The observed and expected number of retroCNVs generated from the X chromosome for each frequency group is provided within the respective bar. The expectation of generating retroCNVs from each chromosome is on the basis of the number of protein-coding genes (with ≥2 exons) within the chromosome. (*B*) Fraction of retroCNVs from each frequency group with insertion site landing in different types of genomic elements. The annotation data of genomic elements in mouse mm10 reference genome is retrieved from Ensembl version 87 (Cunningham, et al. 2019). The statistical significances (*P* values) on the excess of null expectation between X chromosome and autosomes, and the comparisons between pairs of frequency groups, were computed using Fisher’s exact tests. **P value ≤ 0.01; *P value ≤ 0.05; ns: *P* value > 0.05.

To compare these two possibilities, we have categorized retroCNVs into three distinct groups (fig. 2*A*), with singletons (*N* = 977, individual private retroCNVs, i.e., those found only in a single individual) forming the low-frequency group, retroCNVs present in 2–5 individuals forming the intermediate-frequency group (*N* = 552) and present in ≥6 individuals forming the high-frequency group (*N* = 496, note that the latter includes retrocopies that are fixed in given populations).

First, we studied the chromosomal location of parental genes for retroCNVs in the three classes, because it has been suggested that retroCNVs from the X chromosome should have a higher likelihood to have an advantageous effect (Emerson et al. 2004; Schrider et al. 2013), presumably triggered by the compensation of meiotic sex chromosome inactivation (MSCI). The retroCNVs in the singleton group are expected to represent the most recent integration events, thus they can be taken as the baseline of gene retroposition pattern. We found that they show no bias toward coming from genes on the X chromosome, whereas the high-frequency group shows a strong bias and the intermediate frequency group lying between (fig. 2*A*). When a direct comparison between low-frequency retroCNVs and high-frequency retroCNVs was performed, we found a significant excess of gene retroposition coming from the X chromosome (*P* value = 0.02, Fisher’s exact test). This pattern suggests that positive selection may play a role on the retention and increase in frequency of some retroCNVs derived from the X chromosome (see Discussion).

Next, we explored the insertion site pattern of retroCNVs from the three frequency groups, regarding the genomic context of their placement (fig. 2*B*). As expected, most of the retroCNVs are enriched in the intergenic regions, because the non-coding intergenic regions make up the majority of mouse genome sequences. However, the intergenic regions are somewhat less represented for retroCNVs than random expectation, given that they take up >95% of mouse genome sequences (Cunningham et al. 2019). This bias can be primarily explained by the enrichment of low-complexity repetitive sequences in the intergenic regions (Waterston et al. 2002), which reduces the power to detect retroCNVs in our computational discovery pipeline, because it requires one read from given paired-end reads to map to a unique genomic region flanking the retroCNV insertion site (Zhang et al. 2021). It should be noted that such a pattern should not affect the conclusions below, as we were analyzing the propensity of inserting in each type of genetic element for retroCNVs from three frequency groups independently.

Interestingly, we found that high-frequency retroCNVs are more likely to occur in intergenic regions (high-frequency retroCNVs vs. low-frequency retroCNVs: *P* value = 0.008, Fisher’s exact test). This suggests that they are enriched in regions where they do not disrupt other genes, that is, they have a higher likelihood to be neutral or even advantageous. Besides, we observed that intron regions are depleted for high-frequency retroCNVs (high-frequency retroCNVs vs. low-frequency retroCNVs: *P* value = 0.03, Fisher’s exact test), which might be ascribed to the avoidance of retrocopy insertions to disrupt mRNA splicing process (Zhang et al. 2017). With respect to the insertions into coding DNA sequence (CDS) regions, we saw no differences among these three groups, in line with the notion that gene retroposition can be an important mechanism to generate chimeric retrogenes (Casola and Betran 2017).

### Signatures for Positively Selected Gene Retrocopies

To detect direct signatures of positive selection connected to the increases of retroCNV frequency, we focused on retroCNVs of high frequency (≥50%) in all representative populations of a focal subspecies (supplementary table S3, Supplementary Material online), because the positive selection signals of high-frequency retroCNVs are more likely to be stronger and easier for detection. We performed this line of analysis for each subspecies independently (i.e., focusing on subspecies-specific retroCNVs), so as to minimize the influence of confounding factors, such as demographic effects.

Three types of population genetic signals have been reported as associated signatures of positive selection acting on retroCNVs with increased frequency in populations: 1) reduction of average nucleotide diversity (Pi) among nucleotide sequences near the retroCNV insertion site (i.e., selective sweep) for retroCNV carriers in comparison with non-carriers (Schrider et al. 2013); 2) reduced Tajima’s *D* value among nucleotide sequences near the retroCNV insertion site for retroCNV carriers in comparison to background distribution (Llopart et al. 2002); 3) elevated linkage disequilibrium (LD, or *r*^2^) between SNPs near the retroCNV insertion site for retroCNV carriers in comparison with background distribution (Cardoso-Moreira et al. 2016). Herein, we performed rigorous statistical tests on the above three statistics for each focal retroCNV against genomic background distributions (Cardoso-Moreira et al. 2016), which were generated through randomly assigning “pseudo” insertion site of focal retroCNV into genomic regions (see Materials and Methods). We computed the statistical significance as how likely a same or stronger positive selection signature can emerge from the random genomic background distributions, and defined the positively selected retroCNVs as having statistical significances (*P* value < 0.05) for all three statistics.

**Fig. 3.**
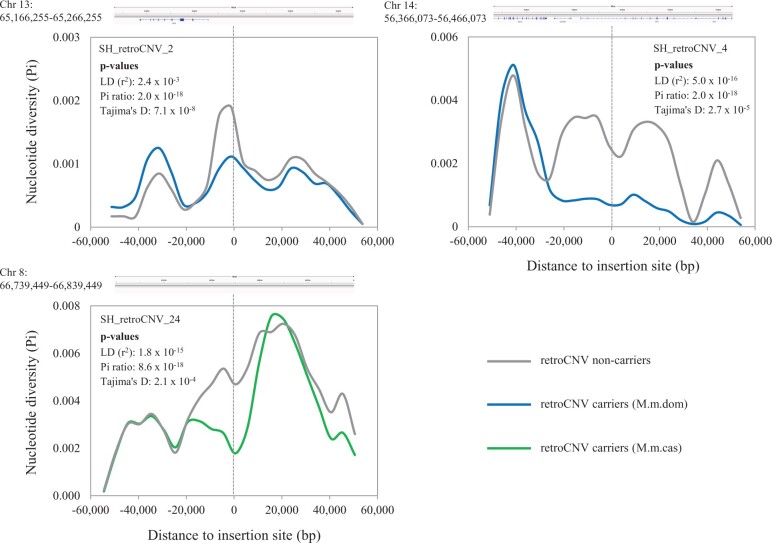
Subspecies-specific high-frequency retroCNVs that show significantly positive selection signatures. Positive selection signature for each retroCNV was tested based on three distinct statistics: 1) reduction of average nucleotide diversity (Pi) among nucleotide sequences near the retroCNV insertion site (i.e., selective sweep) for retroCNV carriers in comparison with non-carriers (Schrider et al. 2013); 2) reduced Tajima’s *D* value among nucleotide sequences near the retroCNV insertion site for retroCNV carriers (Llopart et al. 2002); 3) elevated LD (or *r*^2^) between SNPs near the retroCNV insertion site for retroCNV carriers (Cardoso-Moreira et al. 2016). Only three retroCNVs showing statistical significance (*P* value < 0.05) for all the three statistics compared with random genomic background distributions are presented in this figure. The results on all the tested retroCNVs can be found in supplementary figure S4 and table S3, Supplementary Material online. For each retroCNV, the IGV snapshot in the upper panel shows the genomic context (i.e., annotated genes in the mm10 reference genome) along the 50 kb upstream and downstream flanking region of the retroCNV insertion site. The solid lines in the lower panel represent nucleotide diversity (Pi) within the same flanking region examined in a sliding window of 10 kb and a step size of 5 kb. The dashed gray lines mark the insertion site of retroCNVs. Blue line: *Mus musculus domesticus* individuals of retroCNV carriers; green line: *M. m. castaneus* individuals of retroCNV carriers; gray line: retroCNV non-carriers in corresponding focal subspecies.

Among 18 retroCNVs eligible for such tests (supplementary table S3, Supplementary Material online), we found three with significantly positive selection signatures based on the above criteria (fig. 3). Given the preselection of high-frequency retroCNVs that may show strong positive selection signals, these results suggest that most high-frequency retroCNVs could be neutral or have only moderate positive selection effects on their populations.

### RetroCNVs as Population Differentiation Markers

**Fig. 4.**
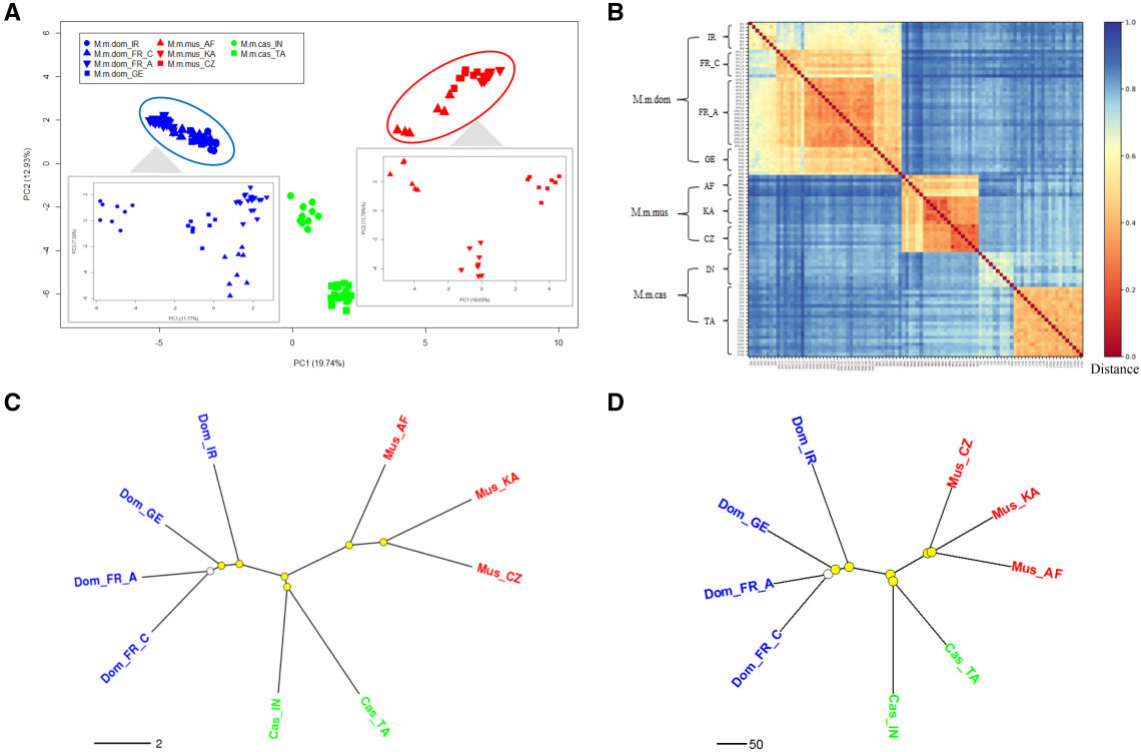
Correlation between the retroCNV landscape and population differentiation. (*A*) Projection of the top two PCs of retroCNV variation in house mouse individuals. Enlarged insets represent the analysis results focusing on populations from the subspecies of *Mus musculus domesticus* and *M. m. musculus* separately, because they cannot be well distinguished in the main figure. (*B*) Heat map on the distance between pairwise individual comparison on retroCNV landscape of all sampled house mouse individuals. The distance between pairwise individuals was defined as the fraction of different retroCNVs of the two individuals to be compared. (*C*) and (*D*) show phylogenetic trees built on the basis of the presence fraction spectrum of retroCNVs within each population and SNP variants that were called from the same genomic data set, respectively. Split nodes marked in yellow are the ones with bootstrap support value >80%. Abbreviations for geographic regions: IR, Iran; FR_C, France (Central Massif); FR_A, France (Auvergne-Rhône-Alpes); GE, Germany; AF, Afghanistan; KA, Kazakhstan; CZ, Czech Republic; IN, India; TA, Taiwan.

The patterns of retroCNVs in natural populations are shaped by a combination of purifying selection (Zhang et al. 2021) and positive selection forces, as shown above. Hence retroCNVs that show elevated population-specific frequencies can be predicted to be more effective markers for recent population differentiation than neutral SNPs (Lee et al. 2014). We set out to test this prediction in our data. First, pairwise individual comparison on the sharing of retroCNVs showed indeed a more similar retroCNV landscape for individuals from the same subspecies/population (supplementary fig. S5, Supplementary Material online), indicating the potential of retroCNVs to infer population relationships. Second, in the overall principal component analysis (PCA), we observed clear clusters of individuals from each mouse population, including the closely related ones when analyzed by subspecies (fig. 4*A*). Similarly, heat map plotting based on the pairwise comparison of individual retroCNV landscapes resolves the populations in defined clusters according to their origin (fig. 4*B*). Finally, on the basis of the presence fraction spectrum of retroCNVs within each population, it is possible to resolve a phylogenetic tree of house mouse populations (fig. 4*C*), for which most of the split nodes are of high confidence (bootstrap support value >80%). The only one exception is the split node between the two French populations, because they are geographically adjacent to each other. The phylogenetic tree is mostly consistent with the one built with the same computational protocol based on the SNP variants that were called from the same genomic sequencing data set (fig. 4*D*), but the nodes of the retroCNV tree are better resolved as predicted. This is most evident for the relationship between the three *M. m. musculus* populations, where the SNP data would suggest a more basic position of the CZ population and a sister group relationship between the KA and AF populations, but with only a poor resolution. The retroCNV data, on the other hand, resolve this clearly and place the AF population more basic, which is also in line with previous conclusions of AF being ancestral and CZ being derived (Hardouin et al. 2015). We conclude that retroCNVs can serve as effective genomic markers to differentiate natural populations better than SNP markers.

Given their effectiveness as population markers, we then performed a retroCNV frequency-based approach to detect the retroCNVs as population differentiating markers (see Materials and Methods). Each retroCNV was assigned a population differentiation index (PDI), to indicate its power to differentiate populations. After applying a false discovery rate (FDR) threshold of 0.05, we detected in total 1,800 retroCNVs (88.6% of all retroCNVs) that could differentiate house mouse natural populations (with significantly larger PDI values compared with null distribution from 1,000 times random shuffling; see supplementary table S1, Supplementary Material online).

## Discussion

Gene copy number variation represents a major source of genetic divergence, and has been shown to play a role in the population differentiation in natural populations of the house mouse (Scavetta and Tautz 2010; Pezer et al. 2015). However, as a particular type of gene copy number variation, gene retrocopies have been missed in previous studies of copy number variation (Schrider et al. 2011). Consequently, their contributions to the adaptation and population differentiation remain largely unknown. Our study represents such a direct analysis to address this question in natural populations of the house mouse. Here we present a comprehensive study of retrocopy dynamics in natural population samples encompassing several lineages and subspecies. We had previously shown that many new retrocopies have deleterious effects in these populations, thus contributing significantly to the overall mutational load (Zhang et al. 2021). Here we explore the retrocopies that are not quickly purged, and we find that they contribute to new adaptations in the respective populations.

### Source of Gene Retroposition

In order to generate heritable retrocopies, gene retroposition events need to occur in the germline, thus it was suggested that genes with higher expression in the germline tend more likely to be the source of gene retroposition (Kaessmann et al. 2009). In support of this prediction, our analysis indeed showed a ubiquitous expression of retroCNV parental genes in the testis and ovary. This is further supported by the observation that retroCNV parental genes being recurrently retroposed to generate multiple retrocopies have a significant higher expression level than the ones that are retroposed once only. These findings therefore suggest that the propensity of a gene to serve for a retrocopy may be a simple function of the amount of mRNA present in the germline.

Intriguingly, these retroCNV parental genes were also found to be highly expressed in all tested somatic tissues. In fact, these genes are likely house-keeping genes involved in a variety of biological processes for the maintenance of the cellular function and thus are probably subject to general transcription in all cells, which is in accordance with the observation of the source of parental genes that are retroposed to generate processed pseudogenes detected in the mouse reference genome (Zhang et al. 2004). This conclusion is strengthened by the finding that retroCNV parental genes are enriched in evolutionarily older gene groups, which tend more likely to be broadly expressed house-keeping genes (Zhang et al. 2015).

### Positive Selection Acting on New Gene Retrocopies

An intriguing pattern on the chromosomal traffic of gene retroposition has been observed with respect to the X chromosome, which has generated a disproportionately high number of fixed functional retrogenes (called “out-of-X” pattern) in mammalian genomes (Emerson et al. 2004; Carelli et al. 2016). It was proposed that this could be related to MSCI, where genes on the X and Y chromosomes are transcriptionally silenced in male germ cells. But retrocopies of house-keeping genes from the X chromosome are expressed in this phase and may therefore partially compensate the silencing of the X-chromosomal genes in the postmeiotic phase of sperm development (Kaessmann et al. 2009). Hence, the observed “out-of-X” pattern would therefore not be due to a higher propensity of X-chromosomal genes to become retrogenes, but due to a higher potential for adaptive retention of these genes (Emerson et al. 2004).

Our data confirm this assumption, as we did not find evidence for a strong initial bias in the generation of new gene retrocopies by parental genes from the X chromosome. However, there is a tendency among the ones occurring at higher frequency to have derived from the X chromosome, supporting the notion that more positively selected ones are among them.

The action of positive selection on some retrocopies is further supported by identifying direct positive selection signatures for three subspecies-specific high-frequency retroCNVs, making them candidates for a particularly strong adaptive process in the respective populations. The agreement between the elevated LD in the flanking regions and the high frequency of these retroCNVs suggests that these retroCNVs are under direct positive selection (Cardoso-Moreira et al. 2016). Moreover, we found no SNPs or copy number variants (CNVs) within the 50 kb up/downstream flanking regions that show complete LD (*r*^2^ = 1) to these retroCNVs. This finding further supports these retroCNV as the direct target of positive selection. Still, it should be noted one cannot completely rule out the possibility from the effects of partial linkage of these variants or other undetected polymorphisms. Hence, we only interpret these findings as suggestive evidence to show the adaptive signatures of these retroCNVs. All three of the parental genes for these gene retrocopies are located on autosomes. They code for a transcription factor interacting protein (*Atf7ip2*), an enzyme (Apobec2) and a vesicle protein (*Vta1*), none of which was so far implicated in specific evolutionary processes.

Our results also show that the evolutionary fate of gene retrocopies is somewhat associated with the genomic context of their placement. The retroCNVs landing in the intergenic regions have a higher probability to increase their frequencies in populations, whereas those ones occurring in the genic regions are restricted to lower frequency. Even though a low fraction of the latter ones may evolve adaptive function such as generating chimeric retrogenes through landing in the exon regions of existing genes (Casola and Betran 2017), the majority of them can be expected to interfere the function of host genes (Schrider et al. 2013). The retroCNVs occurring in the intergenic regions, on the other hand, tend more likely to be neutral or even advantageous, and consequently have a higher likelihood to reach fixation than those ones in the genic regions. All together, these findings suggest that positive selection may drive the spread of retroCNVs in populations, and these retroCNVs might play a role in the adaptation process of natural populations of the house mouse.

### Contribution of Gene Retrocopies on Population Divergence

Given that the patterns of retroCNVs in natural populations are shaped by a combination of purifying selection (Zhang et al. 2021) and positive selection forces as shown in this study, it is reasonable to assume that the retroCNVs could serve as more effective markers to trace the evolutionary history of natural populations in better phylogenetic resolution than the widely used mostly neutral SNPs (Lee et al. 2014). Even though a few attempts have been made to explore gene retrocopy variation in natural populations, so far the analyses of the contribution of gene retroposition on population differentiation were only performed at the level of retroCNV parental genes due to the low resolution to detect gene retrocopy alleles (Abyzov et al. 2013; Zhang et al. 2017). However, in case of inserting into different genomic regions, distinct gene retrocopies can be generated from gene retroposition events from the same parental genes. Owing to the sufficient resolution to call insertion sites of retroCNVs with the unique population genomic data set (Zhang et al. 2021), our study allows a direct analysis to explore the contribution of gene retrocopies to population divergence in the house mouse.

Indeed, we found that gene retrocopies could be efficient markers to infer population relationships, and to resolve the phylogenetic tree of house mouse natural populations, even with better performance than the commonly used SNP variants. We further found that most of the retroCNVs could differentiate house mouse natural populations. Overall, we conclude that the retroCNVs per se can be applied as effective genomic markers to trace the divergence and differentiation history of natural populations.

## Materials and Methods

### RetroCNV and SNP Variant Data Sets

The data set of house mouse lineage-specific retroCNVs was retrieved from our previous study (Zhang et al. 2021). In brief, an optimized computational pipeline combining both exon–exon and exon–intron–exon junction read mapping strategies was exploited to identify gene retroposition events occurring in 96 surveyed mice individuals from nine natural populations of the house mouse (*M.musculus*), whereas absent in both outgroup species (*M.spretus* and *M.spicilegus*). Following the “gold standard” for calling novel retrocopies (i.e., with detectable genomic insertions [Richardson et al. 2014]), we only focused on the house mouse lineage-specific retroposition events with mapped insertion site (Table “All data” of Dataset S3 in Zhang et al. [2021]), corresponding to 2,025 unique house mouse lineage-specific retroCNV alleles that are derived from 1,372 unique parental genes (supplementary table S1, Supplementary Material online) (Zhang et al. 2021). Because the full-length sequences of most gene retrocopies are not available (i.e., those are absent in the mm10 reference genome), we did not separate the ones with intact open reading frames from those not.

We obtained the data set of house mouse lineage-specific SNP variants generated in the same study (Zhang et al. 2021), for which the SNP variants were called by analyzing the same genomic sequencing data set for the detection of gene retroposition events, based on the general GATK version 3 Best Practices (Van der Auwera et al. 2013). Similarly, we only kept the SNP variants with unambiguous ancestral states in out-group species (i.e., same homozygous genotype for all tested individuals from two out-group species), whereas with alternative allele in house mouse individuals for analysis in this study.

### Feature Characterization of RetroCNV Parental Genes

We characterized the features of retroCNV parental genes from three different aspects: 1) gene expression pattern across multiple tissues; 2) GO functional term enrichment; and 3) gene age distribution pattern.

First, we downloaded the raw Illumina paired-end RNA-Seq fastq data for *M.musculus* C57BL/6 inbred line generated by the lab of Michael Snyder in Stanford (Lin et al. 2014), from the ENCODE portal (Davis et al. 2018) (https://www.encodeproject.org/; last accessed December 28, 2021). This data set contains strand-specific transcriptomic sequencing data for 13 tissues dissected from 10-week-old mice (supplementary table S4, Supplementary Material online), including 2 germline tissues (ovary and testis), and 11 somatic tissues (adipose tissue, adrenal gland, brain, heart, kidney, liver, lung, pancreas, sigmoid colon, small intestine, and spleen). We trimmed these fastq files by using Trimmomatic (v0.38) (Bolger et al. 2014), and only kept paired-end reads passed filtering process for further analyses. We mapped the trimmed RNA-Seq reads to mm10/GRCm38 reference genome sequences with HISAT2 (v2.1.0) (Kim et al. 2015), taking advantage of the mouse gene annotation in Ensembl v87 by using the—ss and—exon options of the hisat2-build. Then we counted the fragments mapped to the annotated genes with featureCounts (v1.6.3) (Liao et al. 2014). Finally, we calculated the expression level in FPKM (Fragments Per Kilobase of transcript per Million mapped fragments) for each annotated coding gene within each tissue on the basis of gene’s transcript length and the number of mapped fragments in the data set from each tissue. The expression patterns of retroCNV parental genes were compared with those of all protein-coding genes (with ≥2 exons) annotated in Ensembl v87 as a control. Given the sample size imbalance between retroCNV parental genes (*N* = 1,372) and all protein-coding genes with multiple exons (*N* = 20,500), we performed a bootstrapping analysis for the latter gene group, by randomly sampling the same number of genes as that in the former group for 100 times. The average fractions of genes with expression and standard errors were then computed for all protein-coding genes with multiple exons based on the random sampling data sets. Additionally, we also compared the expression levels between retroCNV parental genes that were retroposed once only and those ones retroposed recurringly (i.e., with ≥2 insertion alleles in the dataset). We utilized Wilcoxon rank-sum tests to compute the statistical significances on the differences of the fraction of genes with expression (FPKM > 0) and gene expression levels.

Second, we performed GO enrichment analysis on retroCNV parental genes with DAVID (Database for Annotation, Visualization, and Integrated Discovery [DAVID] Bioinformatics Resources 6.8) (Jiao et al. 2012). Only significant GO BP (biological process) terms after multiple testing corrections (FDR < 0.05) were kept for presentation.

Lastly, we analyzed the evolutionary gene age pattern of these retroCNV parental genes. Two independent mouse protein-coding gene age assignment data set were downloaded from GenTree (http://gentree.ioz.ac.cn/download.php; last accessed December 28, 2021) (Shao et al. 2019) and (Neme and Tautz 2013), respectively. In brief, each mouse protein-coding gene was dated and given phylostratum/branch assignment by inferring the absence and presence of orthologs along the phylogenetic tree, while using different computational approaches in two data sets. The expected number of retroCNV parental genes in each age group was calculated on the basis of the proportion of protein-coding with at least two exons in this group, because single-exon genes were excluded for the detection of retroCNV alleles in our previous study (Zhang et al. 2021). The fraction of excess of observed retroCNV parental genes for each age group was computed as the deviation between observation value and expectation value divided by the value of expectation within the group. This analysis was performed for each gene age group separately.

### Chromosomal Pattern Analysis

All these 2,025 retroCNVs were classified into three roughly similar-sized groups based on their frequencies in all sampled house mouse individuals: low-frequency group (frequency = 1; *N* = 977), intermediate-frequency group (frequency between 2 and 5; *N* = 552), and high-frequency group (frequency ≥ 6; *N* = 496).

We computed the fraction of excess of observed retroCNVs generated from X chromosome and autosomes for each frequency group. The expected number of generated retroCNVs from each chromosome was calculated based on the number of protein-coding genes (with ≥2 exons) in the focal chromosome. The fraction of excess of observed retroCNVs for each frequency group was computed as the deviation between observation value and expectation value divided by the value of expectation within the focal frequency group, and the statistical significance (*P* value) on the excess within each frequency group was calculated via Fisher’s exact tests. Additionally, we also calculated the statistical significances on the excess of retroCNVs generated from X chromosome between pairs of frequency groups by using Fisher’s exact tests.

### Insertion Site Pattern Analysis

We analyzed and compared the overlap pattern between insertion sites of retroCNVs from the above frequency groups and genomic elements, including intergenic region, protein-coding gene region, intron region, exon region, CDS region, and UTR region annotated in Ensembl version 87 (Cunningham et al. 2019). The “Protein coding gene” category included the whole gene regions, that is, the exon and intron regions. The “Intergenic region” category included the rest of the genomic regions not covered by these protein-coding genes. We required that the “Intron,” “Exon,” “CDS,” and “UTR” categories only come from protein coding gene regions.

Following the convention in Zhang et al. (2017), we enlarged the insertion intervals to 500 bp, that is, 250 bp upstream and downstream from the middle point of the estimated insertion site range. We counted the number of genomic elements by using BEDTools v2.26.0 (Quinlan and Hall 2010), if there was at least 1 bp overlap between the insertion intervals and the genomic elements. We computed the statistical significances on the fractions of retroCNVs landing in each type of genomic element between pairs of frequency groups by using Fisher’s exact tests.

### Positive Selection Signal Detection Analysis

We refined a subset of 27 subspecies-specific high-frequency retroCNVs, by keeping those ones present in at least 50% of mice individuals for all respective populations of the focal subspecies (supplementary table S3, Supplementary Material online). This data set includes 9 retroCNVs in *M. m. domesticus*, 13 retroCNVs in *M. m. musculus*, and 5 retroCNVs in *M. m. castaneus.* Only retroCNVs that are both present and absent in at least three mice individuals for respective subspecies were included, and retroCNVs without SNP variants detected in the flanking regions of the insertion site were excluded for analysis. As a result, 18 retroCNVs were kept for the following analysis (supplementary table S3, Supplementary Material online). All SNPs located within 50 kb upstream and downstream of the insertion sites (middle point) for the mice individuals with and without presence of the retroCNVs in the focal subspecies were extracted separately for further analysis by using VCFtools v0.1.14 (Danecek et al. 2011), based on the above SNP dataset (Zhang et al. 2021).

We first calculated three statistics for each retroCNV by using VCFtools v0.1.14 (Danecek et al. 2011): 1) the ratio of average nucleotide diversity (Pi) along 50 kb up/downstream flanking regions between retroCNV carriers and non-carriers (Schrider et al. 2013); 2) average Tajima's *D* along 50 kb up/downstream flanking region for retroCNV carriers (Llopart et al. 2002); 3) average LD (*r*^2^) along the 5 kb up/downstream flanking region for retroCNV carriers (Cardoso-Moreira et al. 2016). Then we generated the genomic background distribution by assigning “pseudo” insertion site of focal retroCNV into random genomic regions (avoiding sequencing gaps annotated in the mm10 reference genome) for 100 times, and keeping the same setup of retroCNV “carrier” and “non-carrier” individuals. The random insertions were assigned to autosomes only, because all the tested retroCNVs are landing into autosomes (supplementary table S3, Supplementary Material online). We further calculated the three above statistics for each “pseudo” retroCNV by following the same procedure above. The statistical significance (*P* value) for each statistic was computed by using Wilcoxon rank-sum test, through checking how likely to get the same or stronger positive selection signatures (i.e., identical or smaller Pi ratio; identical or smaller Tajima's D; identical or greater LD) from these “pseudo” retroCNV insertions as that from true retroCNV insertion. As a result, we found three retroCNVs of positive selection signals with statistical significance (*P* value < 0.05) for all three statistics. For visualization, we calculated and plotted the average nucleotide diversity levels within the 50 kb up/downstream (in a sliding window size of 10 kb and a step size of 5 kb) of the insertion site of each retroCNV for carriers and non-carriers separately (fig. 3 and supplementary fig. S4, Supplementary Material online).

Additionally, with the SNPs and CNVs called from the same set of individual genomes (Zhang et al. 2021), we searched for the variants that are within 50 kb up/downstream flanking regions of these three retroCNVs and show exactly the same distribution pattern as that of the focal retroCNV (i.e., complete linkage: *r*^2^ = 1). We did not find such SNPs or CNVs for any of these three positively selected retroCNVs.

### Correlation Analysis between RetroCNV Pattern and Population Divergence

We explored the association between retroCNV pattern and population differentiation by conducting three lines of analyses as following: 1) Calculation and comparison on the fractions of shared retroCNVs between all possible pairwise combinations from all individuals, individuals from different subspecies, from different populations but same subspecies, and from the same populations. 2) PCA on the individual retroCNV landscape with R package “ggfortify” v0.4.8 (https://cran.r-project.org/web/packages/ggfortify/index.html; last accessed December 28, 2021). Due to the close relationships among individuals from natural population in *M. m. domesticus* and *M. m. musculus*, we ran additional PCAs for these two subspecies separately. 3) Calculation of the distance between pairwise individual comparison on retroCNV landscape, on the basis of the fraction of different retroCNVs of two individuals to be compared. The heat map plotting of the distance measurement was conducted by using python package “matplotlib” v3.0.1 (https://matplotlib.org/; last accessed December 28, 2021).

### Building Phylogenetic Trees of House Mouse Natural Populations

We constructed the phylogenetic tree for nine natural populations in the house mouse by using neighbor-joining tree estimation function implemented in the R package “ape” v5.3 (Paradis and Schliep 2019), on the basis of the fraction of retroCNV presence spectrum in each population. We used Euclidean distance as the distance measure between each pair of populations. To assess the certainty of the above built phylogenetic tree, we performed 1,000 bootstrap replications (Efron et al. 1996) with boot.phylo function implemented in R package “ape” v5.3. Population split nodes with at least 80% bootstrap support value generated from multiscale bootstrap resampling were taken as ones of high confidence.

To assess the performance of retroCNVs to trace evolutionary history of house mouse natural populations, we built the phylogenetic tree with the aforementioned SNP variants that were called from the same genomic sequencing data set for the detection of gene retroposition events (Zhang et al. 2021). To reduce computation complexity, we performed LD pruning on the SNP data set by using PLINK v1.90b4.6 (Purcell et al. 2007), removing one of a pair of SNPs with LD ≥0.2 in sliding window of 500 SNPs and step wise of 100 SNPs. For each of the remaining SNPs, the fraction of individuals with derived SNP variant (without distinguishing homozygous or heterozygous status) was calculated for each population. We then followed the same procedure to construct the phylogenetic tree for the filtered SNP variants, as it was done for the analysis of retroCNVs.

### Detection of Population Differentiation RetroCNV Markers

We exploited a retroCNV frequency-based approach to detect population differentiating retroCNVs, similar to that was used for analysis of retroCNV parental genes (Zhang et al. 2017). Each retroCNV was assigned a PDI, a measure equivalent to the fixation index (Holsinger and Weir 2009), with the following equations:

(1) $$\mathrm{PDI}=\frac{P(1-P)-\sum_{i=1}^{N}F_{i}*P_{i}*(1-P_{i})}{P(1-P)},$$

(2) $$F_{i}=\frac{N_{i}}{\sum_{i=1}^{N}N_{i}}.$$

Here, *N* represents the total number of natural populations (*N* = 9), *P* is the frequency of a given retroCNV in the individuals from all populations, and *P_i_* is the frequency in the *i*th population of the same retroCNV. Equivalent to the relative population size, *F_i_* is calculated as the number of individuals of the *i*th population (*N_i_*) divided by the total number of individuals from all populations. The significance of PDI for each retroCNV was tested by a randomization test in which we randomly shuffled individual labels for 1,000 times, whereas keeping the same population sizes. The *P* value was calculated by comparing the observed PDI for each retroCNV against the PDI values from the above randomized null distribution. In total 1,800 retroCNVs after multiple testing corrections (FDR ≤ 0.05) were taken as population differentiation markers (supplementary table S1, Supplementary Material online).

## Supplementary Material

Supplementary data are available at *Molecular Biology and Evolution* online.

## Supplementary Material

msab360_Supplementary_Data
