# Supplementary material for: Tracing the Origin and Evolutionary Fate of Recent Gene Retrocopies in Natural Populations of the House Mouse
Source: Mol Biol Evol. 2021 Dec 23;39(2):msab360. doi: 10.1093/molbev/msab360 (PMC8826619; doi:10.1093/molbev/msab360)
Supplement: msab360_Supplementary_Data [file msab360_supplementary_data.zip › Supplementary_Materials_Final.docx]

**Tracing the origin and evolutionary fate of recent gene retrocopies in natural populations of the house mouse**

Wenyu Zhang and Diethard Tautz

Department of Evolutionary Genetics, Max Planck Institute for Evolutionary Biology, August-Thienemann-Str. 2, D-24306 Plön, Germany

**Supplementary Figures:**

**Figure S1** Features of retroCNV alleles

**Figure S2** Comparison of gene expression levels between retroCNV parental genes that are retroposed once only and those retroposed recurringly

**Figure S3** Fraction of excess of retroCNV parental genes based on another independent gene age assignment dataset

**Figure S4** Positive selection results for all subspecies-specific high-frequency retroCNVs

**Figure S5** Fraction of shared retroCNVs for pair-wise individual comparison

**Supplementary Tables (each in separate .xlsx file):**

**Table S1** The information of house mouse lineage-specific retroCNV alleles

**Table S2** GO functional enrichment results of retroCNV parental genes

**Table S3** The information of the subspecies-specific high-frequency retroCNVs

**Table S4** The information of RNA-Seq dataset used in this study

**Supplementary Figure S1**


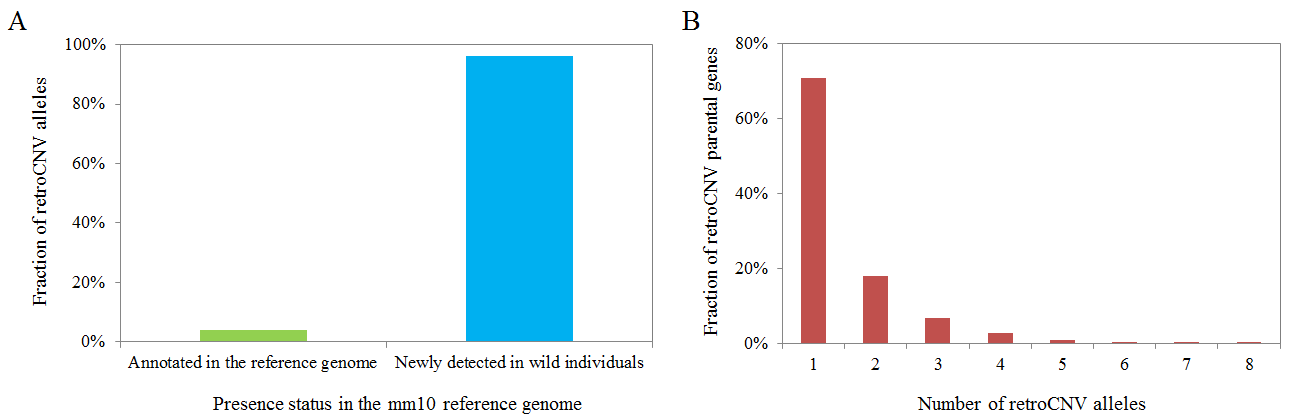


**Supplementary Figure S1:** Features of retroCNV alleles. (A) Fractions of retroCNV alleles with and without annotation in the mm10 reference genome; (B) Fraction of retroCNV parental genes with distinct numbers of detected retroCNV alleles. The calculations are based on the data in the Supplementary Table S1. The annotation data of gene retrocopies in the mm10 reference genome is from RetrogeneDB V2 (Rosikiewicz, et al. 2017).

**Supplementary Figure S2**


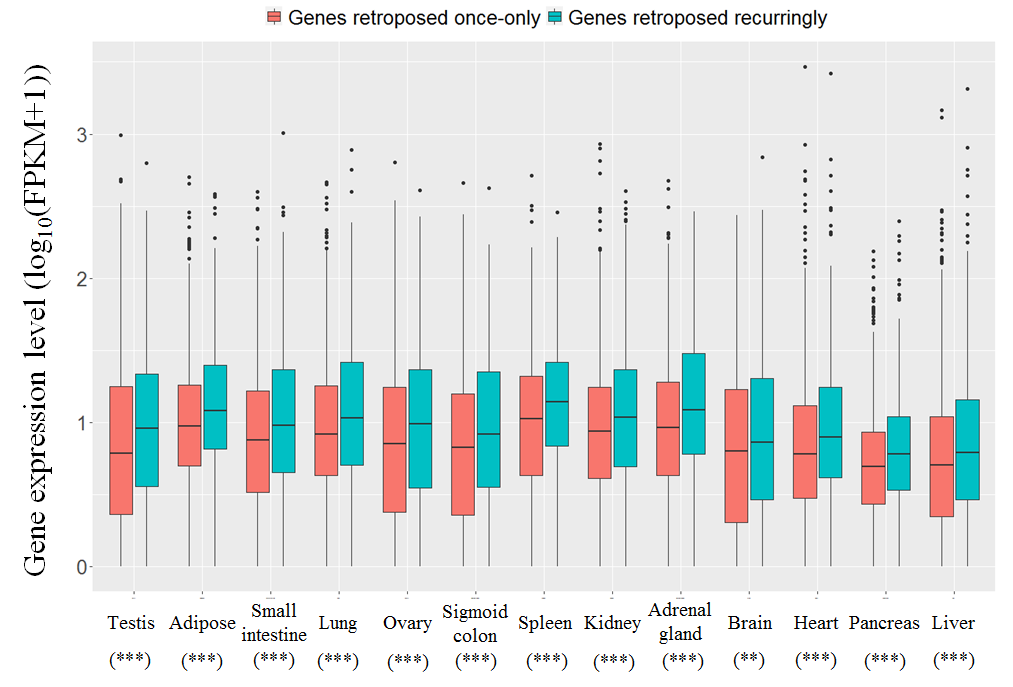


**Supplementary Figure S2:** Comparison of gene expression levels between retroCNV parental genes that are retroposed once only and those retroposed recurringly. The order of tissues in horizontal axis follows Figure 1. The statistical significances were computed with Wilcoxon rank sum tests. ***: p-value ≤ 0.001; **: p-value ≤ 0.01.

**Supplementary Figure S3**


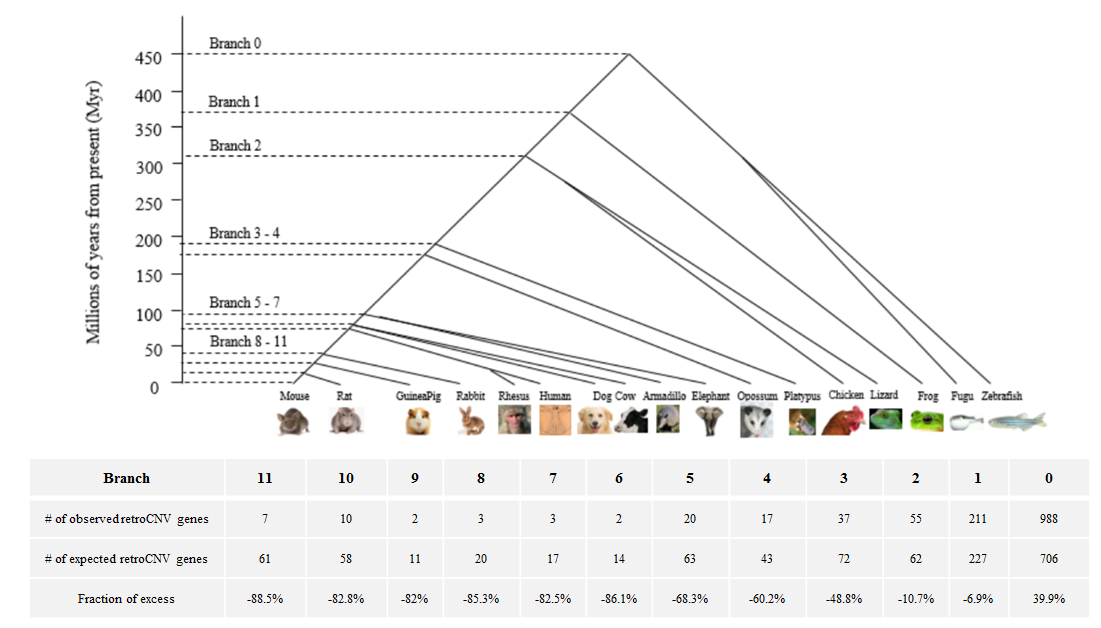


**Supplementary Figure S3:** Fraction of excess of retroCNV parental genes based on another independent gene age assignment dataset. The upper panel shows phylogenetic tree of vertebrates towards mouse together with branches and divergence times in millions of years from present. The phylogenetic branch (age group) assignment data for mouse protein coding genes are retrieved from (Shao, et al. 2019). Larger branch number indicates younger gene age group. The lower panel shows the information on the fraction of excess for retroCNV parental genes within each phylogenetic branch. The null expectation was calculated on the basis of the distribution of annotated protein coding genes with at least two exons within each phylogenetic branch.

**Supplementary Figure S4**


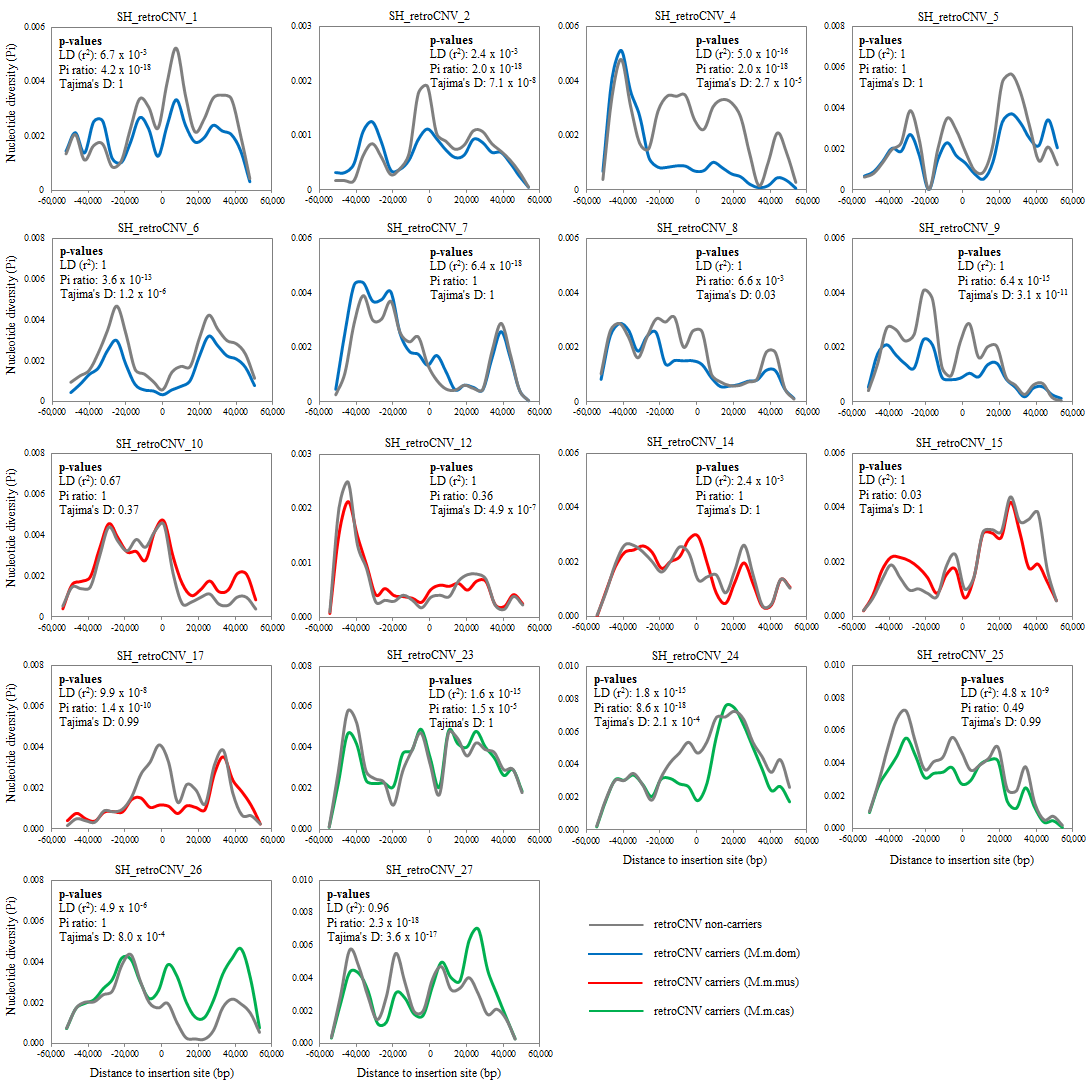


**Supplementary Figure S4** Positive selection results for all subspecies-specific high-frequency retroCNVs. Positive selection signature for each retroCNV was tested based on three distinct statistics: i) reduction of average nucleotide diversity (Pi) among nucleotide sequences near the retroCNV insertion site (*i.e.*, selective sweep) for retroCNV carriers in comparison with non-carriers (Schrider, et al. 2013); ii) reduced Tajima’s D value among nucleotide sequences near the retroCNV insertion site for retroCNV carriers (Llopart, et al. 2002); iii) elevated linkage disequilibrium (LD, or r^2^) between SNPs near the retroCNV insertion site for retroCNV carriers (Cardoso-Moreira, et al. 2016). For each retroCNV, the statistical significance (p-value) for each statistic was computed compared to random genomic background distributions. Solid lines represent nucleotide diversity (Pi) within the 50kb upstream and downstream flanking region of the retroCNV insertion site examined in a sliding window of 10kb and a step size of 5 kb. Blue line: *M. m. domesticus* individuals of retroCNV carriers; Red line: *M. m. musculus* individuals of retroCNV carriers; Green line: *M. m. castaneus* individuals of retroCNV carriers; Grey line: retroCNV non-carriers in corresponding focal subspecies.

**Supplementary Figure S5**


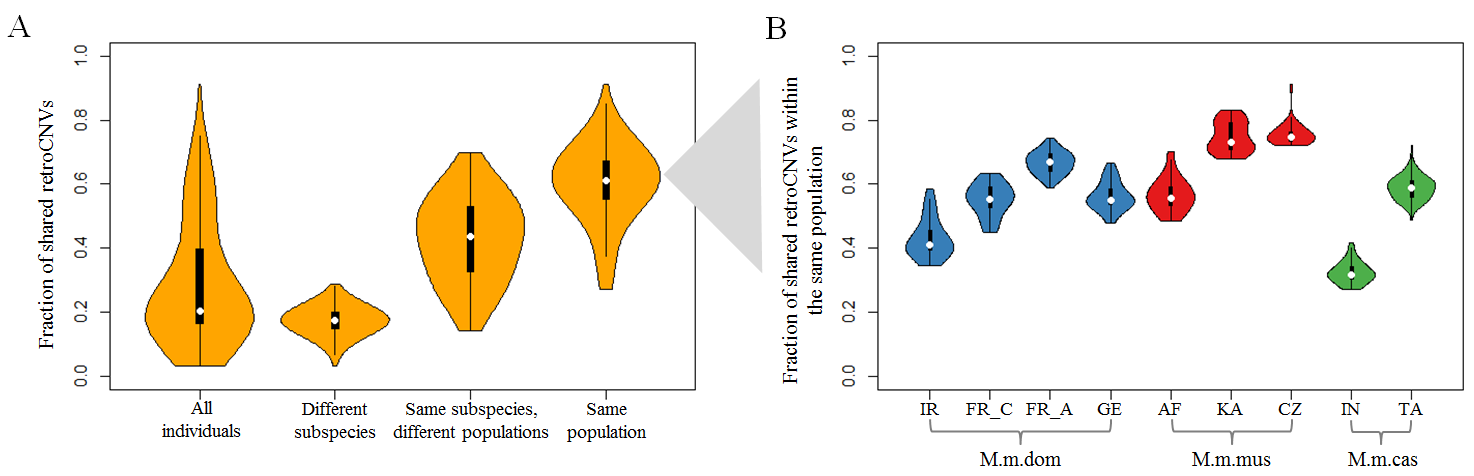


**Supplementary Figure S5** Fraction of shared retroCNVs for pair-wise individual comparison. The fraction of shared retroCNVs between each pair of individuals is defined as the number of overlapping genes divided by the average number of detected retroCNVs of the two individuals to compare. The low fraction of sharing of retroCNVs for Indian population may attribute to a more geographically diverged sampling scheme (Harr, et al. 2016). Abbreviations for geographic regions: IR, Iran; FR_C, France (Central Massif); FR_A, France (Auvergne-Rhône-Alpes); GE, Germany; AF, Afghanistan; KA, Kazakhstan; CZ, Czech Republic; IN, India; TA, Taiwan.

**Reference**

Cardoso-Moreira M, Arguello JR, Gottipati S, Harshman LG, Grenier JK, Clark AG. 2016. Evidence for the fixation of gene duplications by positive selection in Drosophila. Genome Res 26:787-798.

Harr B, Karakoc E, Neme R, Teschke M, Pfeifle C, Pezer Z, Babiker H, Linnenbrink M, Montero I, Scavetta R, et al. 2016. Genomic resources for wild populations of the house mouse, Mus musculus and its close relative Mus spretus. Sci Data 3:160075.

Llopart A, Comeron JM, Brunet FG, Lachaise D, Long M. 2002. Intron presence-absence polymorphism in Drosophila driven by positive Darwinian selection. Proc Natl Acad Sci U S A 99:8121-8126.

Rosikiewicz W, Kabza M, Kosinski JG, Ciomborowska-Basheer J, Kubiak MR, Makalowska I. 2017. RetrogeneDB-a database of plant and animal retrocopies. Database (Oxford) 2017.

Schrider DR, Navarro FC, Galante PA, Parmigiani RB, Camargo AA, Hahn MW, de Souza SJ. 2013. Gene copy-number polymorphism caused by retrotransposition in humans. PLoS Genet 9:e1003242.

Shao Y, Chen C, Shen H, He BZ, Yu D, Jiang S, Zhao S, Gao Z, Zhu Z, Chen X, et al. 2019. GenTree, an integrated resource for analyzing the evolution and function of primate-specific coding genes. Genome Res 29:682-696.
